# Government roles in regulating medical tourism: evidence from Guatemala

**DOI:** 10.1186/s12939-018-0866-1

**Published:** 2018-09-20

**Authors:** Ronald Labonté, Valorie A. Crooks, Alejandro Cerón Valdés, Vivien Runnels, Jeremy Snyder

**Affiliations:** 1 Globalization and Health Equity Research Unit, Faculty of Medicine, University of Ottawa, Ottawa, Canada; 2 Department of Geography, Simon Fraser University, Burnaby, Canada; 3 Department of Anthropology, University of Denver; Denver, Denver, CO USA; 4 Faculty of Health Sciences, Simon Fraser University, Burnaby, Canada; 5 School of Epidemiology and Public Health, University of Ottawa, Alta Vista Campus, 600 Peter Morand Crescent, Room 205A, Ottawa, ON K1G 5Z3 Canada

**Keywords:** Medical tourism, Guatemala, Regulation, Health equity

## Abstract

**Background:**

Regulation of the medical tourism and public health sectors overlap in many instances, raising questions of how patient safety, economic growth, and health equity can be protected. The case of Guatemala is used to explore how the regulatory challenges posed by medical tourism should be dealt with in countries seeking to grow this sector.

**Methods:**

We conducted a qualitative case study of the medical tourism sector in Guatemala, through reviews and analyses of policy documents and media reports, key informant interviews (*n* = 50), and facility site-visits.

**Results:**

Key informants were critical of the absence of effective public regulation of the emerging medical tourism sector, noting several regulatory gaps and the importance of filling them. These informants specifically expressed that: 1) The government should regulate medical tourism in Guatemala, thought there was disagreement as to which government sector should do so and how; 2) The government has not at this time regulated the medical tourism sector nor shown great interest in doing so; and 3) International accreditation could be used to augment domestic regulation.

**Conclusions:**

The intersection of domestic and international regulation of medical tourism has been largely unexplored. This case study advances new research in this area. It highlights the need for and dearth of regulatory protections in Guatemala and lessons for other, similarly situated countries. National regulatory models from Israel and Barbados could be adapted to the Guatemalan context. Global governance could help to protect national governments from any competitive disadvantages created by regulation. Underlying the concerns over growth in medical tourism, however, is how it contributes to the ongoing privatization of health care facilities worldwide. This trend risks undermining efforts to reach targets for Universal Health Coverage and exacerbating existing inequities in the global distribution of health and wealth.

## Background

Medical tourism remains an industry that is predominantly a commercial response to a demand or health care driven by consumers. We use the term ‘medical tourism’ in a somewhat restrictive fashion: the self-selected decision of individuals to seek medical care in a foreign country. We thus exclude from our consideration ill and injured vacationers, expats living much of their time abroad and intermittently needing medical care, and publicly-insured cross-border care initiated or approved by governments, generally due to lack of or undersupply within their own borders. While robust data with regard to medical tourism is hard to come by, the medical tourism industry lays claim to significant growth in recent years, and associated with this growth a promise of economic benefit. This benefit is arguably the key driver of medical tourism on the supply side, and is recognized as such by many countries seeking to develop or expand this offshoring sector [1, 2]. Not only are many national governments supportive of medical tourism, they are also often investors in this sector [3, 4].

Although medical tourism can help to fill unmet health care access needs for citizens of smaller countries lacking higher-end treatment facilities, it has also been criticized for poor safety standards and infection control, and provision of illegal, unethical, or questionable procedures [5–7]. This raises health and safety concerns as well as ethical ones; as a 2013 United Kingdom (UK) study noted, there is “currently no guidance or regulation on risk or safety for UK residents who consider travelling abroad for treatment” [8]. Others point out that there is a “lack of robust clinical governance arrangements and quality assurance procedures in provider organisations” [9]. A decade ago the situation was summed up succinctly: regulation to protect medical tourists’ health is needed [10]; while governments offering such services need to regulate the industry “to ensure the net effects for their [own] citizens is positive” [11]. Regulation here, and as we use the term throughout this article, refers to any form of enforceable government measure “designed to influence business or social behaviour” extending to rules governing “all forms of social or economic influences” [12]; as well as to voluntary or self-regulated systems. The primary areas in which medical tourism and governmental (public) health care regulation intersect include:

• Education and regulation of health professionals, with concerns that both of these may be sub-standard in destination countries to those in countries of international patients [13],

• Changes in a destination country’s accreditation systems to accommodate internationally trained providers working in medical tourism facilities could lower qualification standards [14, 15].

• Accreditation of health care facilities, with concerns over inadequate governmental standards, or of government oversight [16] of the over 40 private international accreditation bodies [17].

• Provision of services that are illegal in the international patient’s home country, and/or are unproven or ineffective treatments [18, 19].

• The role of unregulated or self-regulated intermediary medical tourism services, such as medical tourism brokers, on-line sites, or specialized training programs or certifications in medical tourism that exist outside of publicly regulated professions [20].

• Potential lack of legal redress in cases of alleged medical malpractice, where destination countries may have no, or inadequate, means for international patients to pursue claims [21, 22].

• Lack of requirements for the full sharing of medical files/records, either by the international patient on departure, or by the medical tourism provider on the patient’s return [6, 23].

• Poor oversight of the potential spread of extreme drug resistant or antimicrobial resistant infections that international patients may return with, which can then spread to health care facilities in their home country especially if they require follow-up care [24].

• The intersect between national-level policies and regulations (or lack thereof) and international law, notably core human rights obligations that require governments to ensure that privatization of health markets (including those for medical tourism) or actions of third parties (such as medical tourism brokers) do not interfere with the right of national citizens to health care and health-related services [25, 26].

As part of a multi-country study of the health equity impacts of medical tourism in the Latin American and Caribbean (LAC) region, in this article we examine how different stakeholder groups considered government regulation in one of our case-study countries (Guatemala), which has been attempting in recent years to ‘grow’ this health care sector.

We begin this article by providing some context on our case-study country and then outline our study methods. We next present findings from our study pertinent to issues of regulation, touching on many of the themes identified from the broader literature and some of our earlier studies (above). We conclude with some of the transferable implications of the Guatemalan case for other governments growing this offshoring health care sector, before making an argument for the importance of locating future research on the health equity implications of medical tourism (including studies on regulation) within a political economy and global governance framework.

### Context

Guatemala is a poor country, ranking low on the Human Development Index (125th out of 188 countries) [27] with a per capita GNI of $3,790 [28], or US$7,297 when adjusted for purchasing power parity (PPP) [29], a commonly employed international comparator. Although this puts Guatemala in the World Bank’s grouping of lower-middle income countries [28], it has comparatively high levels of inequality and poverty for the Latin American and Caribbean region [30]. Guatemala has a 2013 Gini coefficient for income distribution of 0.59, indicative of grossly inequitable distribution at a scale associated with increased risk of political and economic instability [31].

Guatemala’s poverty and inequality are reflected in the country’s total per capita expenditure on health, which stood at $476 (Intl $) in 2014 [29]. Approximately 17.8% (2014) of government expenditure is on health, a low amount compared against international and regional levels [32], and accounts for just 2.3% of GDP. Private health expenditure, by contrast, accounts for 3.9% of GDP, of which 83.7% is out-of-pocket [33]. Guatemala’s private health sector is noted as being highly unregulated and fragmented while its public health sector is under-resourced with unequally distributed delivery of services [33]. It has a low density of health human resources, with most physicians located in the urban areas of Guatemala City. Indigenous peoples experience higher levels of poverty and lower access to health care than the non-Indigenous population [30, 33].

Although facing issues in ensuring a reasonable level of domestic health care access, Guatemala is also one of the more recent countries in the region to begin promoting its medical tourism sector. Private organizations including hospitals, hotels, health professionals, and others have organized to form medical tourism clusters [34], and have linked to other organizations with interests in trade, tourism, and medical tourism sectors, such as the Guatemalan Association of Exporters (AGEXPORT) [33]. At present the number of medical tourists seeking care in Guatemala is reported to be low, with no facilities as yet focusing solely, or even primarily, on international medical patients; plans for this expansion, however, have been mooted [33]. Many current Guatemalan medical tourists are from the diaspora, with additional patients coming from proximate countries in the region, although medical tourism providers are keen to attract non-diaspora international patients from the United States (US) and Canada. Individual private care facilities, currently serving primarily local patients (insured or, more often, paying out of pocket) are looking to medical tourism to fill unused capacity [35, 36], similar to how medical tourism developed in Thailand [4], and what we noted during site-visits in India [33, 37]. Some newer Guatemalan facilities have business models premised on increasing international patient flows to levels far exceeding current numbers, raising questions about sustainability of the industry [34, 35].

## Methods

This analysis contributes to an exploratory case study that examined potential and realized impacts of the development of medical tourism in the LAC region on health equity (with a focus on public and private health care, health human resources, investment, and domestic government involvement). Our case study included reviews and analyses of policy documents and media reports pertaining to Guatemala’s growing sector [33], which provided detailed contextual information on the country’s health system (reported above), and its limited forays into medical tourism development, but focused extensively on key informant interviews and health care facility site-visits.

Working in partnership with local research collaborators, recruitment of key informants for interviews was undertaken using a purposeful strategy with a focus on identifying information-rich perspectives. Potential participants were identified through media searches, reviews of relevant policy documents, and through the networks of our local non-governmental organization collaborator. Following the design of the multi-country study, we sought target numbers of key informants from each of five sectors identified as having interests in medical tourism: health human resources representatives (e.g. medical educators, health workers) from both public and private universities and health care sectors (*n* = 15), government representatives responsible for promoting medical tourism and/or regulating health systems (e.g. policy officials) (*n* = 15), public sector representatives (e.g. hospital administrators, tourism planners) promoting or potentially impacted by growth in the medical tourism sector (*n* = 15), private sector representatives (e.g., private clinic owners, private investment consultants, medical tourism investors) with economic interest in growth in the medical tourism sector (*n* = 15), and civil society representatives (e.g., community groups, media representatives) engaged in consultations and/or reporting on medical tourism development in terms of its economic or health equity outcomes (*n* = 15). To protect anonymity in the context of a focused study with s small, saturated sample, we do not provide a further breakdown of participants to avoid identifying individuals.

A total of 50 in-person semi-structured interviews were conducted over a seven-month period, June to December 2013. Our background contextual study [33] was used to identify areas were we could probe deeper for elaboration of responses to our interview schedule. Participants were recruited by phone or e-mail as appropriate and were identified following our review of relevant policy and media documents as well as reviews of human resources profiles for publicly-accessible agencies. All interviews were conducted in the cities of Antigua Guatemala or Guatemala City, which are the focus of medical tourism sector development in the country. Interviews lasted 45–90 min and were conducted in Spanish by a research associate and research assistant hired by our local collaborator. The interview guide was semi-structured and pertained a series of questions common across all participants as well as questions that relate to specific areas of domain expertise. Questions elicited stakeholders’ views/perspectives on issues pertaining to the health equity concerns of focus, including on domestic governments’ roles in regulating medical tourism.

All interviews were digitally recorded. Upon completion they were translated into and transcribed in English. Upon completion the interviews being simultaneously transcribed-translated into English. Our on-site collaborators oversaw this process to ensure the integrity of the transcripts, and served as a reference point for the transcriptionists when assistance was needed. Interview texts were thematically coded and analyzed for descriptive findings related to the roles and implications of government in the regulation of medical tourism. Numerous rounds of independent transcript review, coding extract review, and team meetings were conducted in order to confirm the coding scheme, scope of this analysis, and interpretation of the findings.

Qualitative rigour in this study was established through investigators’ familiarity with the Guatemalan context and the interview transcripts. There was team discussion and agreement on the interpretation of the participants’ perspectives presented in this paper. Throughout the study, investigators maintained a critical and reflective perspective on data interpretation in order that investigator knowledge and standpoints did not skew the participants’ perspectives reported in the paper. Team leaders also took multiple steps to share findings and invited critical feedback and commentary from a wide variety of audiences. The study protocol was ethically approved by ethics review boards at both Simon Fraser University, British Columbia, and University of Ottawa, Ontario.

## Results

In our multi-country study of medical tourism in the LAC region, Guatemala is one of the countries that provided considerable data related to issues associated with regulating medical tourism. It is more of a negative case, insofar that our key informants were critical of the current absence of effective public regulation, noting several gaps and the importance of filling them. Some of the regulatory gaps they describe exist within the public health system as well as affecting the private medical tourism sector, suggesting a weak overall health care regulatory regime within the country.

### Government should regulate medical tourism in Guatemala

Informants were in widespread agreement that it was government’s responsibility to regulate medical tourism in terms of accreditation of facilities. Private sector actors and those involved in the industry itself, however, tended to favour self-regulation over government intervention once facilities were approved. Moreover, the question of *which* government sector should be responsible for *what* aspects of regulation remained moot. One government official maintained that overall government responsibility for the regulation of medical tourism should rest with the Ministry of Economy, since medical tourism lies within the private business sector:

…Every private establishment of health and every clinic [offering medical tourism]…should have permission and mercantile registrations if they are big hospitals, and all those types of things that are regulated by the Ministry of Economy. The technical (health) part (is) the responsibility of the Health Ministry and its directorate of regulation, surveillance and control of health, and its department of regulation of health establishments, [and they] should generate all the process of the establishment’s accreditations, and generate permissions so that the establishments can operate, [with] constant supervision and monitoring.

The Ministry of Health thus retains certain regulatory authority over medical tourism relative to professional licensure, facilities accreditation, and monitoring of services for health care quality control. This separation of the business or trade regulatory side of medical tourism from that of the health care regulation, as one private health care provider noted, can advantage medical tourism sector developers in terms of obtaining promotional support from non-health affiliated ministries, such as the Ministry of Economy and the Ministry of External Relations. It was thought inappropriate (if not politically unwise) for a health ministry in a poor country with an underfunded public health system to be seen promoting a private sector system serving wealthier international patients.

Opinion on this division of regulatory responsibilities, however, was itself divided, with one health professional clearly expressing that *“medical tourism is not a physician business, it’s a corporate business”* while another was adamant that *“It [the Ministry of Health] should be regulating the whole system including the private sector.”* Some informants further suggested that the costs of procedures charged to medical tourists should also be regulated by the health ministry.

Regardless of which branch of government should lead on medical tourism regulation, opinion on the importance of such regulation was a frequent refrain. One of the principle reasons was to ensure quality service to international patients, partly because, as this health professional stated, *“someone can come have a surgery with anyone, have a complication and never come back.”* According to this informant, to avoid this the Guatemalan government must have clearer rules as to *“who can and who can’t treat these patients.”* Secondly, as one public health professional voiced a concern expressed by several others, government regulation was important *“not just because of the sanitary [safety/quality] topic, but also because of the taxes.”* As this informant went on to explain, medical tourism needs to be *“done right, for the benefit of the country”*, noting that *“these people [international patients, medical tourism facilities] are taking - actually using - the infrastructure of the country: airport, services, etc.”* Taxation measures, it was argued, were one essential measure that could ensure that medical tourism is giving something back to citizens of the country.

### But there is a lack of effective regulation

While informants made clear the need for government regulation of the medical tourism sector, they also noted Guatemala’s lack of interest in prioritizing regulation: *“This role isn’t and, historically it hasn’t been, a priority for governments.”* Frequent change of officials or ruling parties in Guatemala was also seen as stalling any forward movement on regulatory reform. Informants further reported on the general disorganization of the Guatemalan health system, including weak regulation in the public health sector, *“a total deficiency of regulation”,* with implications for whether, or how well, regulation might extend to the medical tourism sector*.* The poor state of health regulation in Guatemala, generally, prompted one health system expert informant to suggest that it is creating a free-for-all for private health care development, including medical tourism: *“The Ministry has been…weak as to regulating itself in the services it provides and to regulate the private sector in the health field…everyone wins from whatever they want.”*

In response to being asked “is there a possibility for medical tourism to grow without regulation?” another informant drew on examples of other of the country’s regulated industries:

Yes, without a doubt…it would be like the telephone companies or the credit card companies or insurance companies where they have minimum regulations and they do whatever they want. And since we are talking about commercial conditions not the conditions of the services provided or the quality of the services, of course anything can grow here.

This ‘anything goes’ sense of regulatory indifference to or ineffectiveness of private health sector regulation is also seen as an outcome of incoherence within how health regulations come about within the country. As a government official noted:

The legal part that constructs the health system in the country [is] …complicated because… new legislation, policies, and rules get added… sometimes…contrary to rules and regulations that already exist. This generates conflict in the operational part because everyone looks out for themselves and they say ‘the law says this or the law says that.’

As the above quote suggests, Guatemala’s chaotic legislative system creates contradicting rules that can be ignored or selectively invoked, allowing medical tourism to grow unimpeded by the few health care regulations that might apply. Despite some concern expressed about this confusing state of *“over-regulation in some elements,”* there was also an observed lack of regulation in other important components of health care (e.g. training of health human resources, institutional standard setting, and practices) which have direct relevance to medical tourism facilities. As a health system expert informant noted, there is a pressing need for an overhaul of legislation, and subsequent enforcement:

Universities that “produce” human resources for health should come up with a vision and a system for accreditation, regulation, and certification because the tendency in the country is that new schools of medicine and new schools of nursing are opened, but none of them is measured by any existing standards. Because those standards do not exist.

This statement refers to the post-2000 growth in new private medical and nurse training institutions; most physicians still graduate from the public university [33], although competition from private facilities has begun to challenge the public system’s educational dominance.

### Regulation or international accreditation?

Informants implicitly made distinctions between national (governmental) regulation, which would include accreditation of facilities and training programs, and international accreditation, primarily for health care facilities trying to attract international patients and the qualifications of the medical staff working there. Most emphasized the importance of international accreditation as a demonstration of the quality standards of care, which they saw as important in the promotion of medical tourism. As one government official said:

People who come here for the first time on their own, expect more guarantees for the medical service… And the first thing…is the qualification of the medical staff. So the international recognition of the doctors and the institutions are really important, this international recognition is given throughout accreditations, like the Joint Commission [International] for health services, or any other accrediting organization. And this is the first variable that the client from another country will look at.

The high cost of international facility accreditation was also acknowledged, but was seen as a necessary investment to promote sustainable growth in medical tourism, as a health professional working in this sector in Guatemala explained:

If you want to get accredited, you will have to spend some money, you will have to make an investment, but you will have a better clinic, you will have something better to give the people. And this will reflect because people will come more. This way we will be able to grab that market. But if you are not accredited, which is the international norm, how can you do it? [G]etting accredited is very expensive and it takes a very long time [but] the expensive thing is *not* to pay for the [accreditation].

Even accepting that the costs of international accreditation were simply the price of admission into the global medical tourism marketplace, there remained some worry over the implications for health equity within Guatemala itself. As one of our local health professionals cautioned: *“If you focus too much on meeting the international standards, you might leave behind what matters in your own country; because you don’t worry about things that happen here.”*

## Discussion

Our informants considered it government’s responsibility to prepare and coordinate the conditions for medical tourism, including provision of regulatory structures to ensure the quality, safety, and sustainability of this health care sector. Although the Guatemalan government is reportedly interested in supporting medical tourism for economic development purposes, informants expressed concern that its involvement and support for the health care side of the industry was limited. Informants’ views of the government’s roles in public health regulation generally offered little assurance that Guatemala would soon initiate actions in any of the problematic regulatory areas intersecting medical tourism and public health care, as outlined in our Introduction.

Guatemala is not alone in this regard. Findings from our other studies exploring medical tourism sector development in the LAC region have demonstrated similar tension between the potential for regulation and the will to actually regulate, including in Barbados [14], Jamaica [38], St. Lucia [39], and Cayman Islands [40]. Although our Guatemalan informants generally recognized the need for governments to regulate public health care, opinions on regulation of medical tourism were mixed. Government informants argued that private medical tourism facilities should be regulated in the same fashion as public health facilities. Those working in the private medical tourism sector were content with government licensing of their facilities, and fiscal incentives to grow the industry, but otherwise preferred self-regulation. Although some working in this sector believed that international accreditation (e.g. through JCI [Joint Commission International] a private sector accreditation body, [41]), would be an important signal of high quality care, others remained more sceptical of the value of such accreditation.

### National governance (regulatory) models

There are nonetheless national regulatory models that could be adopted in Guatemala, or other countries pursuing medical tourism. One example is Israel, with a mixed public/private system that reportedly receives 30,000 medical tourists annually, mostly from Russia, Ukraine, Eastern Europe and Cyprus [42]. This practice raised domestic complaints commonly heard in other countries developing this industry: that the fees charged international patients are higher than those paid by Israeli citizens, potentially encouraging hospitals to preference such patients over locals. In May 2017, Israel passed legislation to prevent this. Although still wanting to increase medical tourism for the revenues and foreign currency it brings, it has now imposed several regulatory requirements:

• Care for Israelis must not be harmed, and should ideally improve.

• Income generated through medical tourism (including taxation revenues) will be directed to strengthening the public health system.

• Hospitals must develop data monitoring systems for governments to track the economic activities of hospitals providing care to international patients, to ensure revenues are remitted to the state, as well as monitoring the quality of care received by international patients.

• Limits will be set on the number of international patients each hospital can treat based on waiting times and capacities.

• Intermediaries promoting or brokering medical tourism services must register and abide a list of required policies and practices [43].

How successful such regulations prove in achieving their intent is for future study; but prima facie they address many of the regulatory concerns posed by researchers and health care scholars noted in the introduction to this article. Our research in other LAC region countries, notably Barbados, which is in a similarly early phase of medical tourism development to Guatemala [44], has identified other potential regulatory directions. For example, our focus groups with Barbadian lawyers about the legal and regulatory implications of an expanded medical tourism sector in that country showed the importance of taking measures with regard to immigration law, physician licensing, corporate ownership structures, and reputational protection.

A difference between Barbados and Guatemala, however, and between both LAC region countries and Israel, is the capacity of their respective governments to legislate and enforce regulatory measures. There are inevitably costs associated with establishing and funding new regulatory regimes [45]. Such a regime could be financed through taxes on medical tourism (Israel’s policy), but this would then require governments ideologically committed to regulatory reform. This may be difficult for a country like Guatemala, and for two reasons.

First, and notwithstanding the ‘pink tide’ of governments in the late 1990s and early 2000s, and now in some retreat [46], the LAC region spent years under pro-liberalization and pro-privatization structural adjustment programs and largely embraced neoliberal economic policies associated with the past several decades of globalization [47, 48]. It is hard to discuss regulatory reform without addressing the political economy choices governments have made (or had thrust upon them) in the past, and which can strongly condition their future. Second, and paralleling the first, Guatemala is just one of several countries in the LAC region wanting to attract international medical patients. If none of the other countries similarly pursue regulation of the sector along the lines proposed by Israel (which adds costs all round, including to providers), Guatemala’s ‘good behaviour’ (should it adopt regulations like Israel’s) is likely to disadvantage it competitively.

### Global governance of medical tourism

That whole regions are now in competition with each other in medical tourism begs the question: is there a need for, or possibilities of, effective systems of global-level governance to ensure that (a) medical tourists are treated well, (b) local citizens’ health care access improves, and (c) the allocation of economic benefits of medical tourism disproportionately improves the incomes of poorer, marginalized populations, as called for in the 2015 UN Sustainable Development Goals [49]? There is little disagreement amongst academic scholars that regulating medical tourism at a global level would be preferable to a patchwork of national initiatives. The case for international accreditation standards for medical tourism facilities and practice has been well made and a number of prescriptions argued, including oversight by a major international or intergovernmental body [50]. Existing national governance structures and legal frameworks on treatment and care standards would then need to be harmonized with internationally agreed upon quality standards, and enforced and maintained within countries for both public and private providers [51].

There has been some suggestion of a new global governance platform for medical tourism (under UN, World Health Organization, or World Tourism Organization auspices) to develop international codes of practice or a framework convention based on a review of best regulatory practices [52]. Although such an effort might hold normative power, even if crafted as a legal treaty (e.g. as a framework convention) rather than as a voluntary code, it would still lack sanctioning penalties. It would also require a large number of countries to want such a binding governance instrument. Such an effort would also have to grapple with a number of contradictions between existing international treaties. Guatemala, for example, has made health services liberalization and investment protection commitments under a number of different treaties [33], with such commitments contradicting obligations all countries have under international human rights obligations, including the right to health [53]. Although the right to health is also part of Guatemala’s national constitution (and justiciable by its own courts), the fulfilment of this right is undermined by the parlous state of public health financing, on the one hand, and by apparent governmental indifference, on the other, with one of our informants complaining that: *“In reality the State responds to personal interests (rather) than to the right to health…”.*

### Limitations

One important limitation in our study is that many participants were providing forward-looking comments in that medical tourism is nascent and still very limited in Guatemala. In other words, they were discussing anticipated concerns, issues, and benefits as opposed to realized ones. Capturing these forward-looking comments, however, is very useful, as they provide important insight for interventions that can offset anticipated harms; while identifying barriers and enablers to health-equity oriented regulatory regimes.

## Conclusions

Guatemalan attempts to grow its medical tourism sector appears at this time to be largely in a regulatory vacuum. Few if any of the of the health equity concerns raised by existing literature appear have been addressed by the country, with informants not terribly sanguine that they will be in any short term. This does not minimize their recognition of the importance for such regulation, a concern for many of them that includes regulation of the public health sector as well.

The primary role of regulation is to serve the public. In the context of medical tourism, this extends to three primary ‘publics’: the international patient, the citizens of the destination country, and the citizens of the international patient’s source country. As has been noted by others, it is imperative for Guatemala and for other nations engaged in developing this sector, to give medical tourism much needed regulatory attention:

The rapid developments in medical tourism demands have left the policing and legislation behind. It would be imperative for this legislation to catch up in order to protect the vulnerable that are unable to make well-informed research-based decisions [10].

In a global context in which governance of international trade and financial liberalization is strong, and global governance for health is undeveloped, the international trade regime might attenuate some of the policy options national governments can or should be able to pursue to improve regulation of health care within their own borders [54–56].

The intersection of domestic and international regulation of medical tourism has been largely unexplored. We contend that in advancing new research is this area, there is a need for studies and analyses to incorporate a critical political economy approach. A political economy analysis of medical tourism, for example, would draw attention to how global inequalities in wealth and income distribution create incentives for international patients to seek care abroad, often with little regard for its distributional impact on others. This is more obvious in cases of ethically or legally dubious practices (notably organ harvesting and transplantation) where the wealth of one intersects with the poverty of others, and the pecuniary interests of third-party private providers or foreign currency-hungry governments, is creating outcomes that are inequitable by almost any metric. There are more nuanced aspects to the ethics and economics of many other facets of medical tourism (e.g. fertility or surrogacy tourism, abortion tourism, or simply the ‘crowding out’ of general public health care access); but this is not the place to entertain a deep discussion of these concerns.

It is sufficient to issue a caution that underlying medical tourism is the slow privatization of medical care facilities worldwide [57]. Whether directly owned and outside of any public system, or part of the ‘public-private partnership’ models that have been promoted as short-term fixes for governments’ shortfalls in public revenues, the driving force of such privatization is capital accumulation for the shareholders or managerial class of transnational provider corporations or investment funds [58]. What is left unstated is that scarcity in government revenues incentivizing such developments is an outcome of several decades of global tax competition and the privatization of public assets, eviscerating the ability of most governments to provide equitably for public goods such as public health care [47, 58].

Much of the global health policy attention is now focused on Universal Health Coverage (UHC), and likely will be as the Sustainable Development Goals slowly wind down to their 2030 endpoint. UHC remains a problematic construct that has considerably less political teeth than the more assertive 1978 *Alma Ata Declaration on Primary Health Care* that UHC has come to eclipse [59]. More policy time is now spent debating how UHC might be financed to minimize the impoverishing out-of-pocket payments, including for many of the citizens in Guatemala, than how health systems can achieve health equitable outcomes incorporating policy changes affecting not only medical care, but also the social determinants of health [60]. But unless, and until, the upwards distribution of global wealth (global economic product) from public citizens to private owners is reversed, along with the steep and ongoing decline in overall tax rates that governments apply to economic activities, it is unlikely that the medical tourism (or broader health system) regulatory shortcomings in countries like Guatemala will be resolved.
